# PLASMA PROTEOMIC SIGNATURE OF DECLINE IN GAIT SPEED AND GRIP STRENGTH

**DOI:** 10.1093/geroni/igac059.856

**Published:** 2022-12-20

**Authors:** Xiaojuan Liu, Stephanie Pan, Vanessa Xanthakis, Vasan Ramachandran, Anne Newman, Jason Sanders, Thomas Austin, Michelle Odden

**Affiliations:** Stanford University, Stanford, California, United States; Boston University School of Public Health, Boston, Massachusetts, United States; Boston University School of Public Health, Boston, Massachusetts, United States; Boston University School of Public Health, Boston, Massachusetts, United States; University of Pittsburgh, Pittsburgh, Pennsylvania, United States; Vertex Pharmaceuticals, Boston, Massachusetts, United States; University of Washington, Seattle, Washington, United States; Stanford University, Stanford, California, United States

## Abstract

Physical function predicts health-related quality of life. The biological mechanisms underlying declines in physical function with age remain unclear. We examined the plasma proteomic profile associated with longitudinal changes of physical functions measured by gait speed and grip strength in community-dwelling adults. We applied aptamer-based platform to assay 1,161 plasma proteins on 2,871 participants (60% women, aged 76 years) in Cardiovascular Health Study (CHS) in 1992/1993 and 1,550 participants (55% women, aged 54 years) in Framingham Offspring Study (FOS) in 1991-1995. Gait speed and grip strength were measured annually for 6 years in CHS and at cycles 7 (1998-2001) and 8 (2005-2008) in FOS. The associations of individual protein levels (log-transformed and standardized) with longitudinal changes of gait speed and grip strength in two populations were examined separately by linear mixed effect models. Meta-analyses were implemented using random effect models with a Bonferroni correction for multiple testing. We found that plasma levels of 18 and 12 proteins were associated with changes in gait speed and grip strength, respectively (Bonferroni-corrected p < .05). The proteins most strongly associated with gait speed decline were growth/differentiation factor 15 (GDF-15) (uncorrected Meta-analytic p = 1.60E-15), pleiotrophin (PTN) (1.29E-08), and metalloproteinase inhibitor 1 (TIMP-1) (2.02E-08). For grip strength decline, the strongest associations were for GDF-15 (1.39E-07), carbonic anhydrase III (6.60E-07), and TIMP-1 (3.21E-06). Several statistically significant proteins are involved in the alternative complement pathway, extracellular matrix remodeling or immune function. These novel proteomic biomarkers may inform our understanding of the pathophysiology of functional decline.

